# AI-driven neurosurgery with Sora: applications and complexities

**DOI:** 10.1097/JS9.0000000000001962

**Published:** 2024-07-17

**Authors:** Xinjie Zhang, Jiao Wang, Jinhong Li, Jinhai Gou

**Affiliations:** a Department of Pediatric Neurosurgery, West China Second University Hospital, Sichuan University, Sichuan; b Key Laboratory of Birth Defects and Related Diseases of Women and Children (Sichuan University), Ministry of Education, Sichuan; c Department of Epidemiology, College of Preventive Medicine, Third Military Medical University, Chongqing; d Department of Obstetrics and Gynaecology, Centre for Reproductive Medicine, West China Second University Hospital Chengdu, Sichuan; e Sichuan Provincial Key Laboratory of Development and Related Diseases of Women and Children, Chengdu, Sichuan; f Department of Gynecology and Obstetrics, West China Second University Hospital, Sichuan University, Sichuan, People’s Republic of China

*Dear Editor,*

OpenAI released Sora on 16 February 2024. This latest release has opened new doors of advancement in the artificial intelligence (AI) field and further significant consequences for neurosurgery. OpenAI released Sora on 16 February 2024. This latest release has introduced new advancements in AI and further significant consequences for neurosurgery. By combining diffusion models and transformers, Sora can generate realistic scene videos based on textual prompts, positioning it as a potentially transformative tool in the field of neurosurgery^1,2^.

## Technological applications

### Preoperative planning

Sora’s integration into neurosurgery can revolutionize the visualization and planning of complex procedures. By compressing video data into a low-dimensional latent space and modeling it as spacetime latent patches, Sora can generate detailed, high-definition videos of neuroanatomical structures and surgical interventions. This capability enhances preoperative planning and surgical precision^3^.

### Surgical assistance

During neurosurgical procedures, Sora’s real-time data analysis and augmented reality capabilities can significantly enhance surgical outcomes. The model’s ability to simulate real-world physical laws and create immersive environments can be utilized in neurosurgical simulations, providing surgeons with realistic practice scenarios. This improves the accuracy of procedures such as tumor resections, aneurysm repairs, and spinal surgeries by offering detailed, dynamic visual guides and augmenting the surgeon’s situational awareness^1,3^.

## Clinical practice and education

### Training and education

Sora offers significant advancements in the training and education of neurosurgeons. By generating high-quality, realistic videos based on textual case descriptions or surgical procedures, Sora can create virtual training environments and simulated surgical scenarios. These tools allow neurosurgical trainees to practice and refine their skills in a controlled, immersive setting, improving their understanding of intricate neuroanatomy and surgical techniques. Reck-Burneo *et al*.^4^ found that surgical trainees had significantly higher confidence after watching operative videos compared to reading peer-reviewed manuscripts on surgical techniques.

### Patient experience

Sora enhances patient experience by providing clear, detailed visualizations of surgical procedures and expected outcomes. This capability improves patient understanding and engagement during preoperative consultations, postoperative follow-ups, and rehabilitation processes^5^. By offering visually rich explanations, Sora helps build patient trust and satisfaction, which are critical for successful clinical outcomes^2,3^.

## Minimally invasive surgery

Sora’s capacity to generate detailed, high-quality visualizations from text descriptions can significantly enhance the precision and outcomes of minimally invasive neurosurgical procedures. Techniques such as endoscopic surgery, which rely heavily on visual aids, can benefit from Sora’s ability to create realistic, three-dimensional representations of the surgical site. These representations help surgeons better understand the anatomical relationship between the lesion to be resected and the surrounding vessels and nerves. This enhanced understanding can lead to reduced operative times, smaller incisions, and quicker patient recovery periods.

## Neurostimulation and neuromodulation

Sora can play a pivotal role in functional neurosurgery, particularly in procedures involving neurostimulation and neuromodulation. By providing detailed visualizations of neural pathways and target areas, Sora aids in the precise placement of electrodes for deep brain stimulation (DBS) or responsive neurostimulation (RNS). This enhances the efficacy of treatments for conditions like Parkinson’s disease, epilepsy, and chronic pain^1,2^.

## Interdisciplinary research

Sora’s capabilities extend beyond neurosurgery, fostering interdisciplinary research, understanding, and collaboration. Neurosurgeons can work alongside data scientists, AI researchers, and other medical specialists to explore new applications and innovations. This collaborative approach can lead to the development of novel diagnostic tools, therapeutic strategies, and surgical techniques^2^.

## Addressing challenges and limitations

### Access and equity

Ensuring equitable access to Sora in neurosurgery is a significant challenge. Efforts must focus on providing neurosurgical centers, especially in under-resourced regions, with the necessary infrastructure and training to utilize Sora effectively^1,2^.

### Ethical and social implications

As AI technologies evolve, new legal frameworks are needed to address their unique challenges. For the responsible integration of AI like Sora into neurosurgical practice, it is crucial to ensure regulatory compliance and update legal standards to encompass both the capabilities and risks of these technologies.

### Safety and privacy

Robust security protocols and ethical guidelines are essential to protect sensitive medical information. Addressing potential risks, such as the inadvertent generation of unethical or inappropriate content, is vital to maintaining the integrity and reliability of the AI system.

## Future development

The future development of Sora holds significant promise. Advancements in AI, machine learning, and data processing will continue to enhance its capabilities, potentially expanding its applications in neurosurgery and other medical fields^1,2^.

Ultimately, this advancement contributes to improving the overall quality of patient care. However, it is crucial to address the inherent challenges and complexities associated with AI integration in neurosurgery. Doing so will allow us to harness Sora’s full potential and achieve better patient outcomes.

## Ethical approval

Not applicable.

## Consent

Not applicable.

## Source of funding

Not applicable.

## Author contribution

X.Z., J.W., and J.L.: data collection and writing the paper; J.G.: study concept or design.

## Conflicts of interest disclosure

The authors declare no conflicts of interest.

## Research registration unique identifying number (UIN)

Not applicable.

## Guarantor

Not applicable.

## Data availability statement

The data underlying this article will be shared by the corresponding author on reasonable request.

## Provenance and peer review

Not applicable.
